# Oral Immunization with a Live Coxsackievirus/HIV Recombinant Induces Gag p24-Specific T Cell Responses

**DOI:** 10.1371/journal.pone.0012499

**Published:** 2010-09-02

**Authors:** Rui Gu, Anae Shampang, Toufic Nashar, Manisha Patil, Deborah H. Fuller, Arlene I. Ramsingh

**Affiliations:** 1 Division of Infectious Diseases, Wadsworth Center, New York State Department of Health, Albany, New York, United States of America; 2 Center for Immunology and Microbial Disease, Albany Medical College, Albany, New York, United States of America; IGMM CNRS 5535, France

## Abstract

**Background:**

The development of an HIV/AIDS vaccine has proven to be elusive. Because human vaccine trials have not yet demonstrated efficacy, new vaccine strategies are needed for the HIV vaccine pipeline. We have been developing a new HIV vaccine platform using a live enterovirus, coxsackievirus B4 (CVB4) vector. Enteroviruses are ideal candidates for development as a vaccine vector for oral delivery, because these viruses normally enter the body via the oral route and survive the acidic environment of the stomach.

**Methodology/Principal Findings:**

We constructed a live coxsackievirus B4 recombinant, CVB4/p24(73_3_), that expresses seventy-three amino acids of the gag p24 sequence (HXB2) and assessed T cell responses after immunization of mice. The CVB4 recombinant was physically stable, replication-competent, and genetically stable. Oral or intraperitoneal immunization with the recombinant resulted in strong systemic gag p24-specific T cell responses as determined by the IFN-γ ELISPOT assay and by multiparameter flow cytometry. Oral immunization with CVB4/p24(73_3_) resulted in a short-lived, localized infection of the gut without systemic spread. Because coxsackieviruses are ubiquitous in the human population, we also evaluated whether the recombinant was able to induce gag p24-specific T cell responses in mice pre-immunized with the CVB4 vector. We showed that oral immunization with CVB4/p24(73_3_) induced gag p24-specific immune responses in vector-immune mice.

**Conclusions/Significance:**

The CVB4/p24(73_3_) recombinant retained the physical and biological characteristics of the parental CVB4 vector. Oral immunization with the CVB4 recombinant was safe and resulted in the induction of systemic HIV-specific T cell responses. Furthermore, pre-existing vector immunity did not preclude the development of gag p24-specific T cell responses. As the search continues for new vaccine strategies, the present study suggests that live CVB4/HIV recombinants are potential new vaccine candidates for HIV.

## Introduction

The development of an HIV/AIDS vaccine has proven to be elusive in spite of research efforts spanning over a quarter century [1]–[3]. Current vaccine candidates consist of DNA that encodes HIV peptides or proteins, purified proteins or peptides, and recombinant bacterial and viral vectors that express HIV sequences [1], [4], [5], [6]. In addition, combinations of these vaccine candidates have been used in various prime-boost regimens. Because human vaccine trials have not yet demonstrated efficacy [7]–[10], new vaccine strategies are needed for the HIV pipeline. Because HIV infection is a disease of the mucosal immune system with systemic manifestations [11]–[13], new vaccine approaches must induce both mucosal and systemic T and B cell responses. Given that the gastrointestinal mucosa is the primary reservoir for HIV replication [11], [12], vaccine strategies must be able to target the induction of HIV-specific antibody and T cell responses in the gut. One approach to induce immunity in the gastrointestinal mucosa is by oral delivery of suitable vaccines. A well-known example of an effective vaccine capable of inducing immune responses in the gastrointestinal mucosa and in the systemic circulation after oral delivery, is the live attenuated Sabin vaccine for poliomyelitis [14].

We have been developing a new HIV vaccine platform using a live coxsackievirus B4 (CVB4) vector [15], [16]. Like the polioviruses, coxsackieviruses are small RNA viruses belonging to the genus enterovirus of the family Picornaviridae [17]. Enteroviruses are ideal candidates for development as vaccine vectors for oral delivery, because these viruses normally enter the body via the oral route and are able to survive the acidic environment of the stomach [18]. We have identified a CVB4 variant that is avirulent and immunogenic [16]; mice immunized with the avirulent CVB4 variant are protected when subsequently challenged with a virulent variant. The avirulent CVB4 variant has been developed to express foreign sequences as either structural or non-structural peptides. In our evaluation of the size of the insert that can be expressed as a non-structural peptide, we showed that CVB4/HIV recombinants expressing either 35 or 62 amino acids of gag p24 as amino-terminal extensions of the viral polyprotein are genetically stable [16]. The upper size limit of inserts that is compatible with genetic stability is 100 amino acids. We undertook a proof-of-principle study to examine the immunogenicity, in mice, of a CVB4/HIV recombinant that expresses 73 amino acids of the gag p24 sequence. A comparative study of systemic gag p24-specific immune responses after immunization via either an intraperitoneal or an oral route was undertaken. This is the first report to demonstrate that a live CVB4/HIV recombinant can induce systemic gag p24-specific T cell responses after oral immunization.

## Materials and Methods

### Ethics Statement

All animal procedures were approved by the Institutional Animal Care and Use Committee of the Wadsworth Center under protocol 07-146. The Center is in compliance with the Principles for Use of Animals, the Guide for the Care and Use of Laboratory Animals, the Provisions of the Animal Welfare Act, and other applicable laws and regulations.

### Mice

Female BALB/c mice were purchased from The Jackson Laboratory (Bar Harbor, ME) and immunized at the age of 6 to 8 weeks.

### Construction of the CVB4/p24(73_3_) recombinant

**Figure 1:**
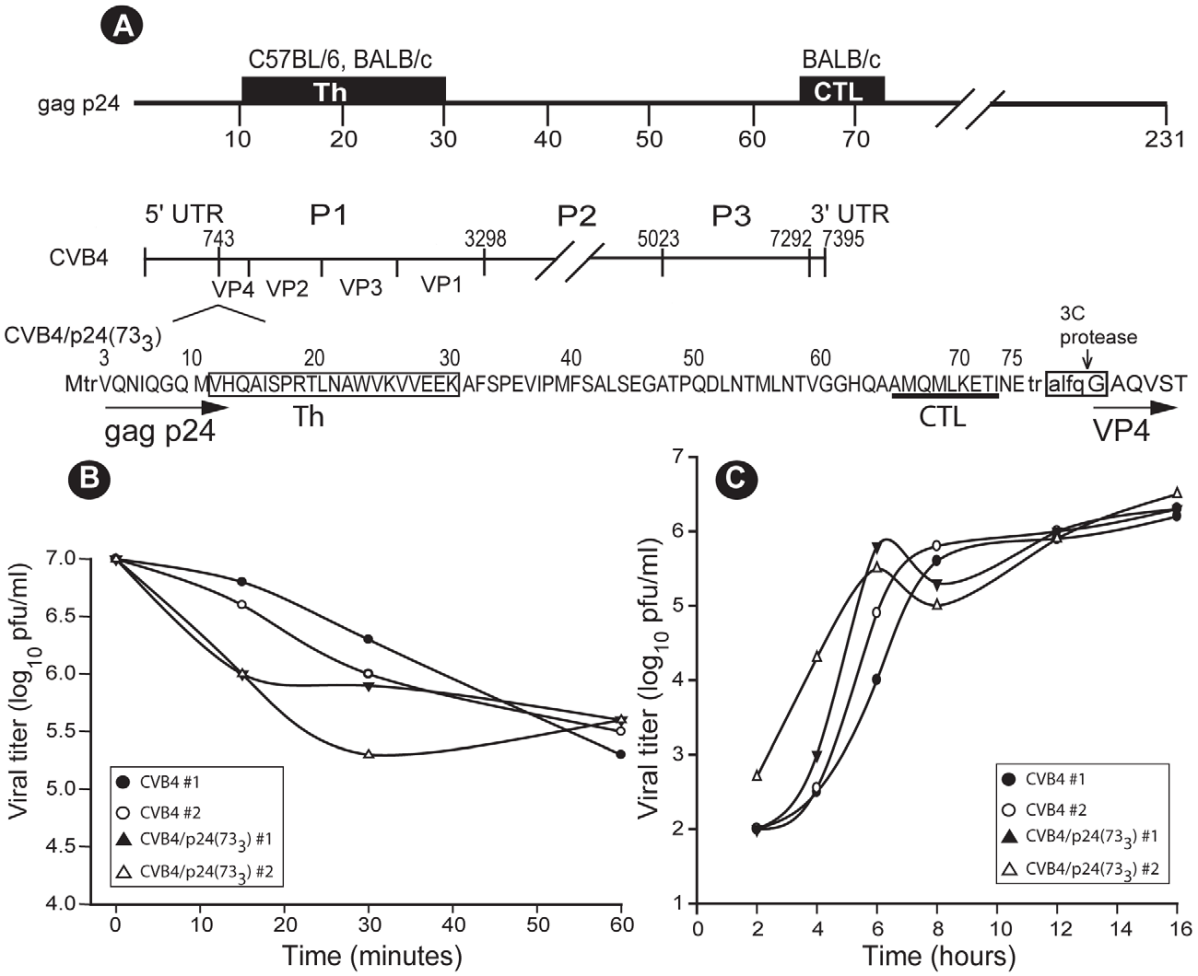
Construction and biological properties of the CVB4/p24(73_3_) recombinant. (A) Construction of the CVB4/p24(73_3_) recombinant. The top line shows the two T cell epitopes of gag p24 that are contained within CVB4/p24(73_3_). The middle line depicts the genetic organization of the CVB4 genome and shows the insertion site for the gag p24 sequence, between the 5′ untranslated region (UTR) and the VP4 sequence. The bottom line shows the 73 amino acids (residues 3 to 75) of gag p24 (HXB2) in the extended polyprotein sequence of CVB4/p24(73_3_). The H-2^d^ restricted T cell epitopes are indicated along with the recognition sequence for the CVB4 3C protease. (B) The CVB4/p24(73_3_) recombinant shares the thermostable phenotype of its parental virus, CVB4. Samples containing 10^7^ pfu of virus were heated at 44°C for different time intervals, and the residual infectivity was determined by plaque assay. Experiments were done twice and both inactivation profiles for each virus are shown. ▴, Δ: CVB4/p24(73_3_); •, ○: CVB4. (C) The CVB4/p24(73_3_) recombinant is replication-competent. The replication of CVB4/p24(73_3_) and CVB4 in LLC-MK2(D) cells was assessed under single-step conditions. Each experiment was done twice and both growth curves for each virus are shown. ▴, Δ: CVB4/p24(73_3_); •, ○: CVB4.

For the CVB4/p24(73_3_) recombinant, the gag p24 sequence (HXB2) corresponding to amino acids 3 to 75 was amplified by PCR from a plasmid, HIV-gpt, and cloned into the CVB4 polyprotein cassette vector as previously described [16]. The gag p24 sequence was positioned immediately after the initiator codon of the VP4 sequence and was followed by a sequence encoding a recognition site for the 3C protease (Fig. 1A). Clones were screened initially by restriction enzyme analysis and verified by DNA sequence analysis. RNA transcripts, synthesized from recombinant cDNA clones with T3 RNA polymerase, were used to transfect LLC-MK2(D) cells by electroporation. Virus was harvested when cells exhibited 80–100% cytopathic effect (CPE). Recombinant viruses were plaque-purified on LLC-MK2(D) cells. To confirm the presence of the gag p24 sequence in the recombinant virus, limited sequence analysis was done both on viral RNA obtained from plaque-purified virus and on RNA obtained from infected cells [19]. RNA samples were reverse-transcribed using random primers and specific regions of the viral cDNA were amplified by PCR and sequenced. Plaque-purified virus was used to prepare a large-scale stock of the CVB4/p24(73_3_) recombinant in LLC-MK2(D) cells.

### Intraperitoneal and oral immunizations of mice

Immunization was by intraperitoneal (IP) injection or by oral gavage. Mice were immunized with either CVB4/p24(73_3_) or the parental CVB4. PBS-treated mice served as controls. For both IP and oral immunizations, primary immune responses were assessed 10 to 14 days after administration of one dose (5×10^4^ pfu) of virus. Secondary immune responses were assessed after the administration of an immunizing dose (5×10^4^ pfu) of virus and a boosting dose (10^6^ pfu) of virus. For IP immunizations, the boost was administered 4 weeks after the immunizing dose, and spleens were harvested at various time points (2, 7, 14, or 28 days) after the boost. Maximal responses were observed 14 days after the boost. For oral immunizations, the boost was administered two-to-four weeks after the immunizing dose and spleens were harvested at various time points (2, 7, 10, or 14 days) after the boost. Maximal responses were observed 10 days after the boost. The difference in the timing of administration of the boost reflects altered viral replication after immunization via the intraperitoneal and oral routes. Intraperitoneal immunization with either CVB4/p24(73_3_) or the parental CVB4 resulted in systemic infection and higher viral replication while oral immunization caused a localized infection and lower viral replication. Pathogenicity was monitored by measurement of body weight, assessment of viral titers in multiple organs, and evaluation of pancreatic tissue damage by histology. For the histological studies, pancreatic tissues were fixed with Bouin's fixative (Sigma-Aldrich, St. Louis, Mo.), processed for routine histology, and stained with hematoxylin and eosin.

### Infectivity assays

Organs were weighed, and homogenates were prepared in PBS using a Mini-BeadBeater (Biospec Products, Bartlesville, OK) and 1.0 mm zirconia/silica beads (Biospec Products, Bartlesville, OK). Three organs per mouse - intestine, pancreas, and spleen - were individually homogenized in 2 ml of PBS. For both Peyer's patches and mesenteric lymph nodes, 5 to 6 nodules per mouse were collected and homogenized in 1.0 ml of PBS. Viral titers in organ homogenates were measured using a standard plaque assay. After oral delivery of virus, infectious virus was not detected by plaque assay in any organ. For detection of low levels of infectious virus early after oral delivery, HeLa cells, grown in 6-well plates, were infected with 0.2 ml of organ homogenate and monitored for the development of CPE.

### Detection of anti-CVB4 antibodies by ELISA

ELISA Maxisorp (Nunc) 96 well plates were coated with 0.25 µg per well of purified coxsackievirus B4 in PBS and incubated at 4°C overnight. Plates were washed three times with PBS and 0.02%Tween 20 and blocked with PBS and 20% FBS for 2 hr at 37°C. Serial-fold dilutions of sera in PBS and 0.02%Tween 20 were loaded into duplicate wells. After incubation for 30 min at 37°C, plates were washed three times and peroxidase-conjugated sheep anti-mouse antibody (Sigma) were added to the wells. Plates were incubated for 30 min at 37°C and then washed three times. The chromogenic substrate, 2,2′-azino-bis-(3-benzthiazoline-6-sulfonic acid) (Sigma), was added and optical densities were measured using an ELISA plate reader Sunrise (Tecan) at 405 nm. Sera from PBS-treated mice served as negative controls.

### ELISPOT assay

The cytokine ELISPOT assay [20] was used to enumerate the number of individual IFN-γ secreting cells in spleens of immunized mice. All anti-mouse antibodies were obtained from BD Biosciences Pharmingen (San Diego, CA) or eBiosciences (San Diego, CA). Briefly, 96-well nitrocellulose-based plates were coated with rat anti-mouse IFN-γ antibody at a final concentration of 10 µg/ml. Plates were incubated overnight at 4°, washed with PBS and blocked with 5% bovine serum albumin (BSA). Splenocytes (2×10^6^ cells/well) were cultured in the presence of an HIV-1 gag p24 peptide (2 µg/ml) for 24 hr at 37°C. The two gag p24 peptides used in the study are p24(10–30) and p24(65–73). The p24(10–30) peptide contains an H-2^d^ restricted T helper cell epitope [21], while the p24(65–73) peptide contains an H-2^d^ restricted CTL epitope [22]. Cultures were done in duplicate. Negative controls consisted of splenocytes cultured with an unrelated peptide (either from ovalbumin or from the circumsporozoite protein of Plasmodium berghei) or no antigen. Positive controls consisted of cells treated with either heat-inactivated CVB4 (1×10^6^ PFU/well) or a combination of anti-mouse CD3 antibody and anti-mouse CD28 antibody. After washing with PBS, biotinylated rat anti-mouse IFN-γ antibody was added to each well. After washing with PBS, the bound biotinylated antibody was detected using a streptavidin-alkaline phosphatase conjugate. The number of spots on each filter was counted using a CTL-ImmunoSpot S5 UV Analyzer (Cellular Technology Ltd, Shaker Heights, OH) and CTL ImmunoSpot software (Cellular Technology Ltd). The negative controls for the IFN-γ ELISPOT assay allowed us to define a negative response as less than 50 spot-forming cells (SFC) per well.

### Multiparameter flow cytometry

HIV-1 gag p24-specific T cell responses in mice immunized with CVB4/p24(73_3_) were assessed using a five-color intracellular staining assay. Splenocytes (10^6^ cells/well) were cultured with either of the two HIV-1 gag p24 peptides (2 µg/ml), p24(10–30) or p24(65–73), for 1 hr at 37°C. Splenocytes from mice immunized with the parental virus, CVB4, and cultured with the HIV-1 gag p24 peptides, served as the vector control. Negative controls consisted of splenocytes from mice immunized with either CVB4/p24(73_3_) or CVB4 and cultured with an unrelated peptide (either from ovalbumin or from the circumsporozoite protein of Plasmodium berghei). Positive controls consisted of splenocytes treated with anti-CD3 (5 µg/ml) and anti-CD28 (2 µg/ml) antibodies.

The BD Cytofix/Cytoperm Plus kit (BD Biosciences, San Diego, CA) was used for inhibition of intracellular protein transport and for the fixation and permeabilization of cells. BD GolgiPlug (0.5 µl/ml), a protein transport inhibitor containing brefeldin A, was added to the cultures which were then incubated for an additional 4 to 5 hr at 37°C. The following rat anti-mouse monoclonal antibodies (BD Biosciences Pharmingen, San Diego, CA) were used: anti-CD4-PE-Cy7, anti-CD8-PerCP, anti-IFN-γ-FITC, anti-IL-2-PE, and anti-TNF-α-APC. Splenocytes were incubated initially with the anti-CD4 and anti-CD8 antibodies on ice for 30 min. Cells were then fixed and permeabilized with the Cytofix/Cytoperm solution. After washing with the Perm/Wash buffer, cells were incubated with the anti-IFN-γ, anti-TNF-α, and anti-IL-2 antibodies on ice for 30 min. After washing in PBS, cells were fixed in 1% paraformaldehyde and analyzed by flow cytometry using the BD FACSAria II (BD Biosciences, San Jose, CA). Gag p24-specific T cell frequencies in CVB4/p24(73_3_) immunized mice were calculated as follows: [T cell freq CVB4/p24(73_3_)_gag p24 peptide_ – T cell freq CVB4/p24(73_3_)_unrelated_ _peptide_] - [T cell freq CVB4_gag p24 peptide_ – T cell freq CVB4_unrelated peptide_].

## Results

### The CVB4/p24(73_3_) recombinant retains the physical and biological characteristics of the parental CVB4 vector

The CVB4/p24(73_3_) recombinant contains an insert of 243 nucleotides of which 219 nucleotides are from the gag p24 sequence (Fig. 1A). Because a longer CVB4 genome could alter the physical packaging of RNA and capsid proteins, thereby affecting viral stability, we evaluated the physical stability of the recombinant. The thermal stability of CVB4/p24(73_3_) was compared to that of the parental virus, CVB4 (Fig. 1B). The parental virus is thermostable: after 1 hr at 44°, infectivity decreased an average of 40-fold. The CVB4/p24(73_3_) recombinant was more rapidly inactivated than the parental vector during the first 30 min of inactivation. However, after 1 hr of inactivation, overall infectivity decreased an average of 25-fold and was thus similar to that observed for the parental virus. The CVB4/p24(73_3_) recombinant shares the thermostable phenotype of its parental virus. To determine whether the insertion of the gag p24 sequence into the CVB4 genome affected viral replication, we analyzed the growth kinetics of CVB4/p24(73_3_) in cell culture, under single-step conditions (Fig. 1C). After 16 hr, CVB4/p24(73_3_) replicated as well as the parental vector. However, the kinetics of replication differed. During the exponential phase of growth, the recombinant replicated to higher titers than the CVB4 vector. We also evaluated the genetic stability of the CVB4/p24(73_3_) recombinant after multiple passages in cell culture, at low multiplicities. Viral sequences from infected cells were reverse-transcribed, amplified by PCR, and sequenced. Deletion products were not observed during amplification (data not shown). After seven passages in cell culture, the recombinant retained the 219 nucleotides of the gag p24 sequence. The CVB4/p24(73_3_) recombinant is thus physically stable, replication-competent, and genetically stable.

### Pathogenicity is influenced by the route of delivery

**Figure 2:**
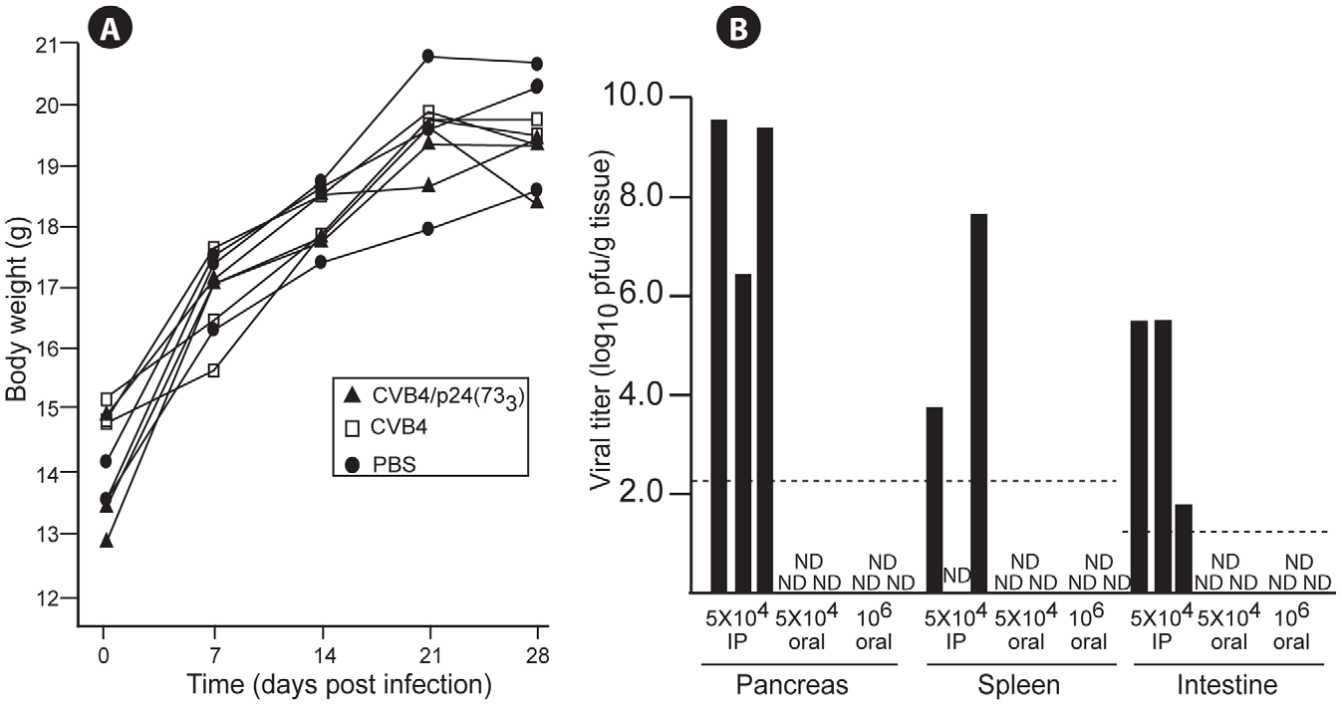
Replication of the CVB4/p24(73_3_) recombinant in BALB/c mice. (A) Mice infected with CVB4/p24(73_3_) gained weight. Mice (n = 3) were infected intraperitoneally with CVB4/p24(73_3_) (▴) or CVB4 (□) or were mock-infected with PBS (•), and body weights were measured at various times after infection. (B) Intraperitoneal delivery of CVB4/p24(73_3_) resulted in systemic infection, while oral delivery did not result in systemic infection. Mice (n = 3) were infected with 5×10^4^ pfu administered intraperitoneally, 5×10^4^ pfu administered orally, or 10^6^ pfu administered orally. Pancreas, spleen, and intestine were harvested from each mouse at 2 dpi, and organ homogenates were tested for viral infectivity by plaque assay. Dashed lines indicate the limit of detection of infectious virus. ND: not detected.

We assessed whether the gag p24 insert affects viral pathogenicity by monitoring CVB4/p24(73_3_) infected mice for visible signs of infection, changes in body weight, viral load in target organs, and pancreatic tissue damage. BALB/c mice infected with CVB4/p24(73_3_) either orally or intraperitoneally appeared healthy, showed no visible signs of infection and gained weight (Fig. 2A). We measured viral loads by plaque assay in organs harvested 2 days after infection with CVB4/p24(73_3_) because viral loads in pancreatic tissues are maximal 2 days after infection via the intraperitoneal route with the parental vector [23]. Infectious virus was detected in pancreas, spleen, and intestine of BALB/c mice infected intraperitoneally with a dose of 5×10^4^ pfu of CVB4/p24(73_3_) (Fig. 2B). Of the three organs, viral titers were highest in pancreas. In contrast, infectious virus was not detected by plaque assay in any of the organs of mice infected orally with either the same dose (5×10^4^ pfu) or a higher dose (10^6^ pfu) of CVB4/p24(73_3_) (Fig. 2B). The data for the higher inoculum were confirmed by monitoring the development of CPE in HeLa cells infected with organ homogenates (Table 1). We next investigated whether infectious virus could be detected in mice earlier than 2 days after oral delivery of the higher dose of 10^6^ pfu of CVB4/p24(73_3_).

**Table 1:** The CVB4/p24(73_3_) recombinant induced a localized infection in the gut after oral delivery.

	No. of organs containing infectious virus				
Time p.i. (hrs)	Intestine	PP*	MLN^#^	Spleen	Pancreas
8	3/3	0/3	0/3	0/3	0/3
16	3/3	1/3	0/3	0/3	0/3
24	3/3	1/3	1/3	0/3	0/3
48	0/3	ND	ND	0/3	0/3

*Peyer's patches (5–6 in each sample);

# mesenteric lymph nodes (5–6 in each sample). Organ homogenates (0.2 ml) were used to infect HeLa cells and infectivity was evaluated by the development of CPE. ND, not done.

We reasoned that low titers of infectious virus might be below the level of detection of the plaque assay and decided to monitor infectivity by the development of CPE in HeLa cells. While infectious virus was detected in the intestine, for all infected mice, from 8 to 24 hr after infection, infectious virus was not detected in spleen or pancreas (Table 1). Infectious virus was absent from the mesenteric lymph nodes at 8 and 16 hr after infection and was detected in only one sample at 24 hr after infection. Infectious virus was also absent in all samples of Peyer's patches at 8 hr after infection and was detected in one sample at 16 hr and in one sample at 24 hr after infection. The combined data indicate that after oral delivery, the CVB4/p24(73_3_) recombinant induces a short-lived, localized infection of the gut without systemic spread.

**Figure 3:**
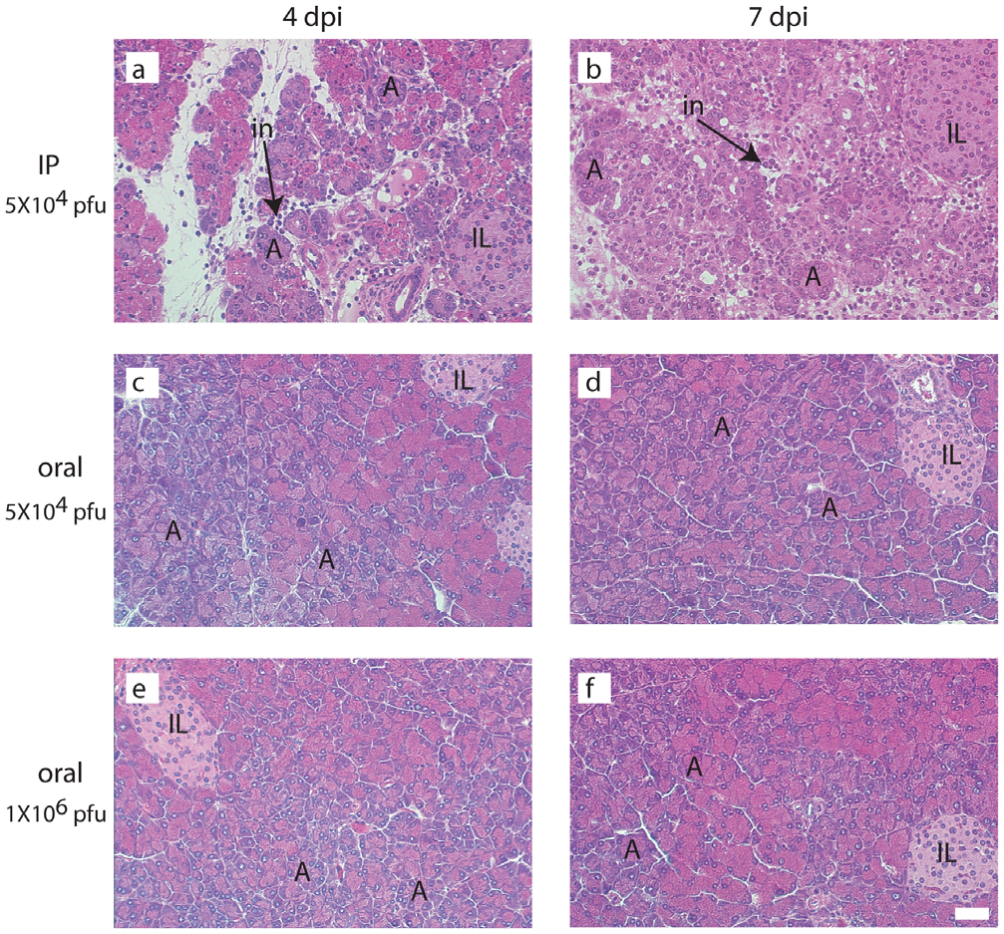
Transient pancreatitis develops after intraperitoneal administration of CVB4/p24(73_3_) but it does not develop after oral administration. Mice (n = 4) were infected with 5×10^4^ pfu administered intraperitoneally, 5×10^4^ pfu administered orally, or 10^6^ pfu administered orally. The pancreas was harvested from 2 mice at 4 dpi and from 2 mice at 7 dpi. Tissues were processed for routine histology and stained with hematoxylin and eosin. Representative sections are shown. (a) 4 dpi, IP infection with 5×10^4^ pfu, depicting pancreatitis; (b) 7 dpi, IP infection with 5×10^4^ pfu, depicting pancreatitis; (c) 4 dpi, oral infection with 5×10^4^ pfu, depicting a healthy, intact pancreas; (d) 7 dpi, oral infection with 5×10^4^ pfu, depicting a healthy, intact pancreas; (e) 4 dpi, oral infection with a higher dose,10^6^ pfu, depicting a healthy, intact pancreas; (f) 7 dpi, oral infection with a higher dose,10^6^ pfu, depicting a healthy, intact pancreas. A, acinus (core structure of the exocrine pancreas consisting of 8-12 acinar cells; IL, islet of Langerhans; in, inflammatory infiltrates. Original magnification, ×250. The scale bar is 25 µm.

While infected mice showed no visible signs of disease, histological assessment of pancreatic tissues revealed a transient pancreatitis in mice that had been infected by the intraperitoneal route although not by the oral route (Fig. 3). BALB/c mice infected intraperitoneally with 5×10^4^ pfu of CVB4/p24(73_3_) developed a transient pancreatitis from 4 dpi to 7 dpi (Fig. 3, panels a and b). Pancreatitis affected approximately 50% of the organ and was characterized by exocrine tissue loss, focal inflammation, and edema. By 14 dpi, pancreatitis had resolved and the exocrine tissues showed complete recovery (data not shown) similar to that observed in mice infected with the parental CVB4 vector [24]. In marked contrast, BALB/c mice infected orally with 5×10^4^ pfu or 10^6^ pfu of CVB4/p24(73_3_) did not develop pancreatitis (Fig. 3, panels c–f). Pancreatic acinar cells were well-granulated and intact and showed no evidence of damage.

### Evaluation of the antigenicity of the CVB4/p24(73_3_) recombinant and the CVB4 vector

**Figure 4:**
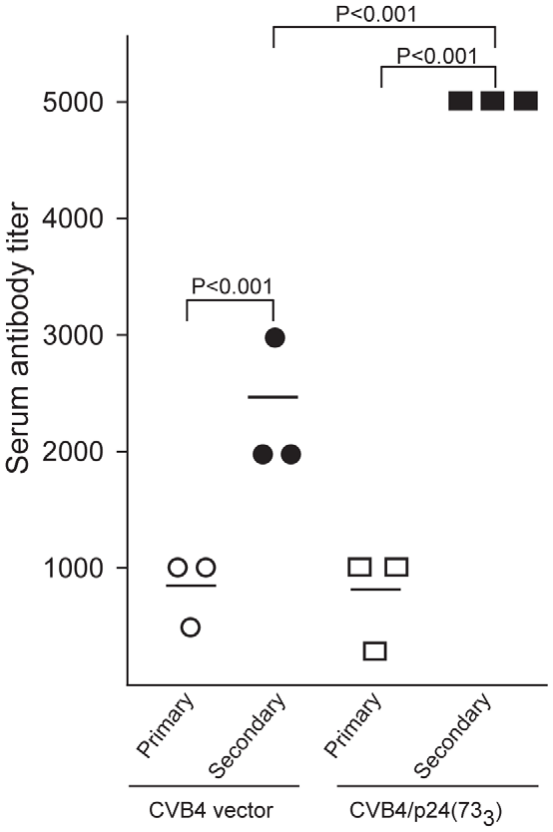
Mice immunized with either the CVB4 vector or the CVB4/p24(73_3_) recombinant develop an anamnestic response to vector antigens. Mice (n = 3) were immunized via the intraperitoneal route with either CVB4 or CVB4/p24(73_3_). Sera from mice immunized with one dose of virus (primary responses) or three doses of virus (secondary responses) were tested for anti-CVB4 antibodies by ELISA. Primary anti-CVB4 titers were similar in mice immunized with either CVB4 (open circles) or CVB4/p24(73_3_) (open squares). Secondary anti-CVB4 titers were greater in mice immunized with CVB4/p24(73_3_) (filled squares) than in mice immunized with CVB4 (filled circles). Statistical analysis was done using a one-way ANOVA.

The CVB4/p24(73_3_) recombinant is designed to express the gag p24 sequence as a non-structural protein and is expected to retain the antigenicity of the parental CVB4 vector. To test whether the antigenicity of the recombinant is similar to the parental vector, mice were immunized intraperitoneally with either CVB4/p24(73_3_) or CVB4. Sera harvested 10 days after either primary or secondary immunization were assayed by ELISA for antibodies that bound to the purified CVB4 vector. Antibody titer is defined as the reciprocal of the lowest serum dilution whose absorbance at 405 nm is greater than four-fold the absorbance of the corresponding dilution of control sera. Primary anti-CVB4 titers were similar in mice immunized with either CVB4 or CVB4/p24(73_3_) (Fig. 4). Both groups of immunized mice showed an anamnestic response to vector antigens because anti-CVB4 titers increased after immunization with more than one dose of virus (Fig. 4). Furthermore, the anamnestic response of mice immunized with CVB4/p24(73_3_) is greater than that of mice immunized with CVB4. It is unclear why the recall response to vector antigens is greater in mice immunized with CVB4/p24(73_3_). Given that CVB4/p24(73_3_) replicated to higher titers than CVB4 during the exponential phase of growth (Fig. 1C), a possible explanation is that the recombinant replicates to higher titers than the vector *in vivo*. Increased viral replication is expected to generate a greater amount of vector antigens, capable of inducing a more vigorous antibody response.

### Assessment of cytokine production from splenocytes of mice immunized with CVB4/p24(73_3_)

**Figure 5:**
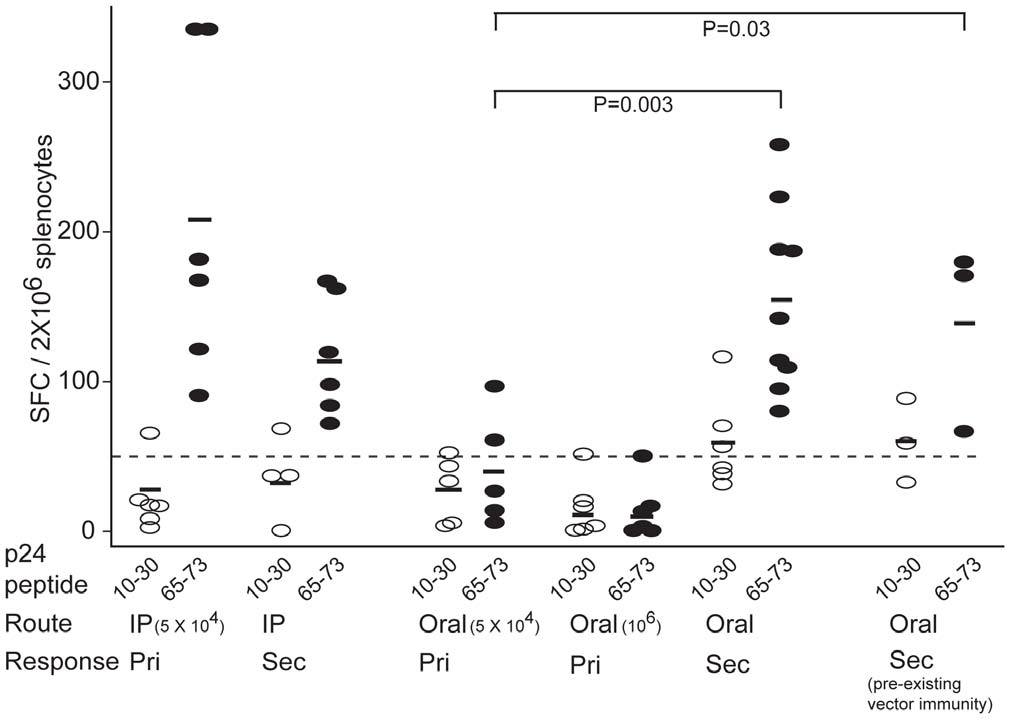
Gag p24-specific IFN-γ production by splenocytes after IP or oral immunization with CVB4/p24(73_3_). Primary (Pri) and secondary (Sec) immune responses were monitored using an IFN-γ ELISPOT assay. The immunizing dose used in these studies was 5×10^4^ pfu, except in one study in which a higher dose,10^6^ pfu, was used to evaluate primary responses after oral delivery. IFN-γ production in response to p24(10–30) is indicated by open symbols while IFN-γ production in response to p24(65–73) is indicated by filled symbols. Each symbol represents the result from one mouse. Negative controls defined a negative response as less than 50 SFC per well, indicated by a dashed line. Gag p24(65–73)-specific primary and secondary immune responses were observed after IP immunization, while gag p24(65–73)-specific secondary immune responses were observed after oral immunization of CVB4 naïve and CVB4 immune mice. Statistical analysis was done using the t-test.

Splenocytes from immunized mice were stimulated with either of two gag p24 peptides, p24(10–30) and p24(65–73), each of which contains an H-2^d^ restricted T cell epitope present in CVB4/p24(73_3_), and IFN-γ production was monitored by the ELISPOT assay. Primary immune responses were monitored after administration of one dose of CVB4/p24(73_3_), whereas secondary immune responses were monitored after administration of two doses of CVB4/p24(73_3_). Splenocytes from mice immunized intraperitoneally with one dose (5×10^4^ pfu) of the recombinant produced IFN-γ in response to stimulation with the p24(65–73) peptide but not in response to stimulation with the p24(10–30) peptide (Fig. 5). Splenocytes from mice immunized orally with one dose (5×10^4^ pfu) of the recombinant failed to produce IFN-γ in response to stimulation with either of the gag p24 peptides. Oral immunization with the higher dose (10^6^ pfu) of CVB4/p24(73_3_) also failed to elicit primary immune responses against gag p24. Unlike primary immune responses, secondary immune responses to the p24(65–73) peptide were detected after either intraperitoneal or oral immunization. A boost effect was observed after immunization by the oral route but not after immunization by the intraperitoneal route. The data suggest that one intraperitoneal dose of the recombinant is sufficient to induce strong primary gag p24-specific immune responses while two oral doses of the recombinant are needed to induce strong secondary immune responses.

Secondary immune responses to gag p24 were also evaluated in mice pre-immunized with the parental vector, CVB4. Because the normal route of transmission for coxsackieviruses is the oral route, mice were pre-immunized orally with CVB4. To verify that mice developed immunity to the CVB4 vector, serum samples, collected 7 days after the pre-immunization, were assayed for anti-CVB4 antibodies by ELISA. Mice pre-immunized orally had high antibody titers (ranging from 500 to1,000) against the CVB4 vector. Vector-immune mice were then immunized orally four weeks later and boosted orally with the CVB4/p24(73_3_) recombinant. As was observed with naïve mice immunized with CVB4/p24(73_3_), CVB4-immune mice immunized with CVB4/p24(73_3_) generated gag p24-specific immune responses. IFN-γ production was detected by splenocytes stimulated with the p24(65–73) peptide (Fig. 5).

### Splenic CD4 and CD8 T cells from mice immunized with CVB4/p24(73_3_) produced cytokines after stimulation with HIV-1 gag p24 peptides

Splenocytes from immunized mice were stimulated with either of the two gag p24 peptides, p24(10–30) and p24(65–73), and T cells producing TNF-α, IL-2, or IFN-γ were identified by multiparameter flow cytometry. The functionality of gag p24-specific T lymphocyte responses was assessed using Boolean gating and FlowJo software (TreeStar Inc., Ashland, OR).

**Figure 6:**
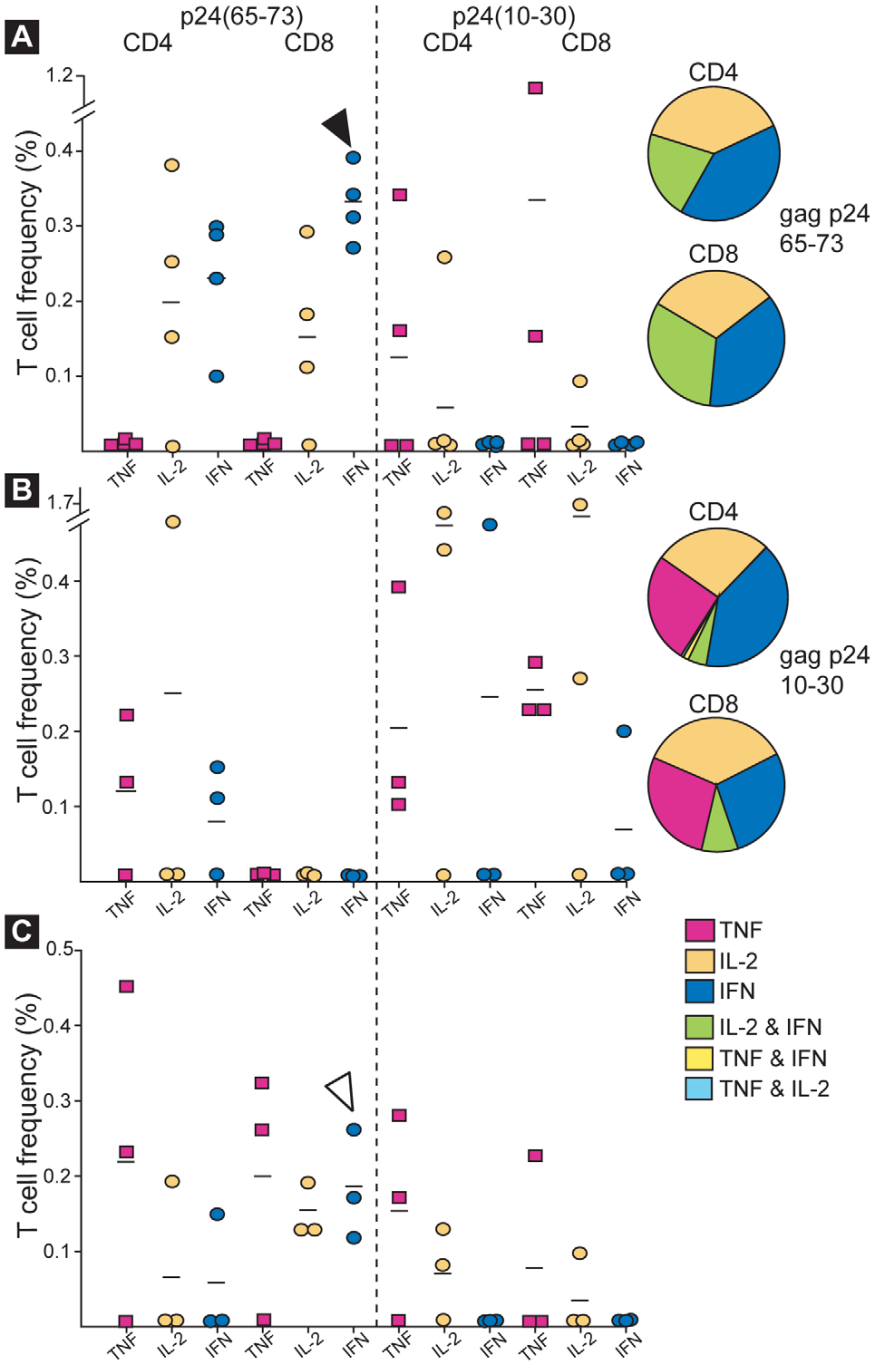
T cells from mice immunized either intraperitoneally or orally with CVB4/p24(73_3_) produce cytokines in response to gag p24 peptides. Splenic CD4 and CD8 T cells producing TNF-α, IL-2, or IFN-γ in response to p24(65–73) or p24(10–30) were identified by multiparameter flow cytometry. (A) Primary immune responses after immunization via the intraperitoneal route. (B) Secondary immune responses after immunization via the intraperitoneal route. (C) Secondary immune responses after immunization via the oral route. Each symbol represents the results from one experiment using splenocytes pooled from 3 mice. Experiments were done a total of three or four times and all of the data are shown here. Mean values are indicated by horizontal bars. Flow cytometric analysis was performed after gating on CD4 or CD8 T cells. The T cell frequency indicates the percentage of CD4 or CD8 T cells that is responding to the gag p24 peptides. T cell frequencies after IP immunization with one or two doses of virus were similar. The one exception is indicated by a closed arrowhead (P<0.05). T cell frequencies after IP or oral immunization with two doses of virus were also similar. The one exception is indicated by an open arrowhead (P<0.05). Statistical analysis was done using a one-way ANOVA. The functionality of gag p24-specific T cell responses was assessed using Boolean gating and FlowJo software, and the data is shown in pie charts. Polyfunctional T cells were detected after IP immunization but not after oral immunization.

A statistical analysis of the frequency of T cells producing cytokines in response to gag p24 peptides reveals that gag p24-specific T cell responses are similar after intraperitoneal immunization with one or two doses of CVB4/p24(73_3_) (Fig. 6A and 6B). The one exception was IFN-γ production by CD8 T cells in response to the p24 (65–73) peptide which was stronger after immunization with one dose of the recombinant. In comparing T cell responses after immunization via different routes, we observed that the frequencies of T cells producing cytokines in response to gag p24 peptides were similar after intraperitoneal or oral immunization (Fig. 6B and 6C). Again, the only exception was IFN-γ production by CD8 T cells in response to the p24 (65–73) peptide which was stronger after oral immunization. The intracellular staining studies support the results of the ELISPOT studies and suggest that one intraperitoneal dose of CVB4/p24(73_3_) is sufficient to induce strong primary gag p24-specific immune responses while two oral doses of the recombinant are needed to induce strong secondary immune responses.

The functionality of gag p24-specific T cell responses was assessed using Boolean gating and FlowJo software, and the data is depicted as pie charts in Fig. 6. Polyfunctional T cells were detected after intraperitoneal immunization but not after oral immunization. Furthermore, immunization with one dose of the recombinant yielded polyfunctional T cells that responded to the p24(65–73) peptide but not to the p24(10–30) peptide (Fig. 6A, pie-charts). In a representative experiment, of the CD4 T cells responding to the p24(65–73) peptide after one dose of the recombinant, 38% produced IL-2 alone, 40% produced IFN-γ alone, and 21% produced both IL-2 and IFN-γ. Of the CD8 T cells responding to the p24(65–73) peptide, 31% produced IL-2 alone, 37% produced IFN-γ alone, and 32% produced both IL-2 and IFN-γ. While immunization with one dose of the recombinant yielded polyfunctional T cells that responded to the p24(65–73) peptide, immunization with two doses of the recombinant resulted in polyfunctional T cells that responded to the p24(10–30) peptide (Fig. 6B, pie-charts). In a representative experiment, of the CD4 T cells responding to the p24(10–30) peptide after two doses of the recombinant, 27% produced IL-2 alone, 41% produced IFN-γ alone, 26% produced TNF-α alone, and 6% produced dual combinations of cytokines. Of the CD8 T cells responding to the p24(10–30), 36% produced IL-2 alone, 28% produced IFN-γ alone, 28% produced TNF- α alone, and 9% produced both IL-2 and IFN-γ. The data indicate that while the frequency of T cells producing cytokines in response to gag p24 peptides are similar after intraperitoneal immunization with one or two doses of CVB4/p24(73_3_), the quality of the responses differ.

The data from the ELISPOT assays show that splenocytes from mice immunized with the CVB4/p24(73_3_) recombinant produced IFN-γ in response to the immunodominant CTL epitope, p24(65–73), but not to the non-dominant T helper cell epitope, p24(10–30) while the data from the flow cytometric studies show that splenic T cells from immunized mice produced cytokines in response to both gag p24 peptides. A possible explanation for this observation is that the p24(10–30) peptide induced lower levels of cytokine per cell than the p24(65–73) peptide. The lower response to the p24(10–30) peptide was detected by intracellular staining because the assay concentrates cytokines in each cell.

## Discussion

We showed that a live recombinant, CVB4/p24(73_3_), expressing 73 residues of gag p24, retained the physical, biological, and antigenic characteristics of the parental vector. Oral immunization with the CVB4 recombinant was safe and resulted in the induction of systemic HIV-specific T cell responses. Furthermore, pre-existing vector immunity did not preclude the development of gag p24-specific T cell responses.

The gag p24 sequence within the CVB4/p24(73_3_) recombinant contains two well-characterized H-2^d^ restricted T cell epitopes, an immunodominant CTL epitope and a non-dominant T helper cell epitope [21], [22]. The CVB4/p24(73_3_) recombinant was designed to express the gag p24 sequence as an intracellular, non-structural peptide which would be processed through the MHC class I pathway, thereby allowing presentation of the p24 CTL peptide to CD8 T cells. Uptake of extracellular antigens from cellular debris by antigen presenting cells (APCs) is expected to allow processing through the MHC class II pathway, thereby allowing presentation of the p24 T helper cell epitope to CD4 T cells [25]. In addition, cross-presentation of extracellular antigens by APCs is expected to amplify presentation of the p24 CTL peptide to CD8 T cells. Data from the five-color intracellular assay showed that CD8 T cells from mice immunized with CVB4/p24(73_3_) did, in fact, produce cytokines after *in vitro* stimulation with the p24 CTL peptide, while CD4 T cells produced cytokines after *in vitro* stimulation with the p24 T helper cell peptide. The data also show that CD8 T cells produce cytokines after stimulation with the p24 T helper cell peptide while CD4 T cells produce cytokines after stimulation with the p24 CTL epitope. The reasons for these observations are unclear. A potential explanation of the CD8 T cell response after stimulation with the p24(10–30) peptide is that a sequence within this T helper cell peptide is also able to stimulate CD8 T cells.

We also evaluated the effect of immunizing mice with the CVB4/p24(73_3_) recombinant using different doses and different routes of delivery. We showed that a boost effect was observed after oral immunization but not after intraperitoneal immunization. In addition, immunization via the oral route or the intraperitoneal route resulted in similar frequencies of gag p24-specific T cells. The one exception was that oral but not intraperitoneal immunization induced CD8 T cells that produced IFN-γ in response to the p24 CTL peptide.

While we have been working on a live CVB4-based HIV vaccine platform, other groups have been developing polioviruses as replicating and non-replicating vaccine vectors. The initial study to show that picornaviruses could be developed as vaccine vectors was done using poliovirus [26]. The poliovirus recombinants induced antibody responses [26] and CTL responses [27] against the inserted sequences. Replication competent poliovirus recombinants, based on the Sabin poliovirus vaccine strains, have also been constructed to express the entire SIV genome in discrete, overlapping fragments [18]. Macaques immunized with the poliovirus-SIV vaccine cocktails showed some protection after challenge with SIV_mac_251 [28]. A non-replicating poliovirus replicon system has also been developed to express HIV-1 and SIV sequences [29]. The poliovirus replicons were immunogenic in macaques. A limitation of the live poliovirus vector system is that immunogenicity studies must be done in macaques because poliovirus does not replicate in wild-type mice. A major advantage of a CVB4-based HIV vaccine platform is that extensive immunogenicity studies can be done in mice. Vaccination strategies for the CVB4/HIV recombinants can be tested and optimized in mice prior to evaluation in macaques. Another picornavirus that has been developed to express HIV-1 sequences is the human rhinovirus 14. A combinatorial approach has been used to construct libraries of rhinoviruses expressing an HIV-1 gp41 epitope [30]. Recombinant rhinoviruses induced modest levels of HIV-1 neutralizing antibodies in guinea pigs.

For any vector system that is under development for use as an HIV vaccine, a key question is whether pre-existing vector immunity will inhibit the ability of the recombinant vaccine to induce HIV-specific T and B cell responses [5], [31]. This concern is particularly relevant for a coxsackievirus vector because coxsackieviruses are ubiquitous in the human population [32]. We have shown that mice immunized via the intraperitoneal route with the avirulent parental CVB4 vector, were protected when subsequently challenged with a virulent CVB4-V variant [16] indicating that pre-existing immunity has an impact on second exposure. Data from the present study further support this observation since mice immunized with a second dose of the CVB4 recombinant, delivered via the intraperitoneal route, did not show increased gag p24-specific immune responses. However, immunization via the oral route was able to by-pass pre-existing immune responses since oral immunization with two doses of the CVB4 recombinant resulted in increased immune responses. We also addressed whether oral immunization with the CVB4 recombinant was able to bypass pre-existing vector immunity. We showed, in a pilot study, that oral immunization with the CVB4/p24(73_3_) recombinant induced gag p24-specific systemic immune responses in mice with pre-existing vector immunity, and these responses were comparable to responses in vector-naïve mice. The data support the conclusion that oral immunization with the recombinant bypasses pre-existing immunity. Vaccine vectors such as adenovirus [33], [34] and Listeria monocytogenes [35] have also been shown to bypass pre-existing immunity when administered via the oral or nasal routes.

Another important issue in the use of recombinant viruses as vaccines is safety. Historically, live attenuated, replicating viruses have provided the most effective protection against disease [5]. By contrast, inactivated or subunit vaccines induce immunity of more limited duration. A driving factor in the development of inactivated or subunit vaccines is safety. Clearly, there is a trade-off between safety and efficacy in developing a viral vaccine. In humans, most infections with the group B coxsackieviruses are asymptomatic [36]. When symptomatic infections occur, the group B viruses cause mild illnesses of the upper respiratory tract, the gastrointestinal tract, and the skin. Rare serious manifestations of acute CVB infection include aseptic meningitis, myocarditis, and pericarditis. A few case reports and studies of animal models suggest links between CVB infections and chronic diseases of the heart [37] and pancreas [38], [39]. Data from studies of CVB pathogenesis indicate that immunomodulatory approaches are beneficial in treating both acute and chronic stages of disease [40], [41]. Should a CVB4-based vaccine be developed, then increased research efforts will be needed to identify appropriate immune-based therapies for treating reversion events.

The CVB4 vector used to construct the CVB4/p24(73_3_) recombinant has been studied extensively, and several reports show that the CVB4 vector induces a transient pancreatitis in mice, after infection by the intraperitoneal route [23], [24], [36], [38], [42]. The present report shows that the pathogenicity of the CVB4/p24(73_3_) recombinant is influenced by the route of infection. Administration of the CVB4/p24(73_3_) recombinant via the intraperitoneal route resulted in a transient pancreatitis, similar to that observed with the parental CVB4. However, administration of the recombinant via the oral route resulted in a short-lived, localized infection of the gut, without systemic spread. Mice immunized orally with CVB4/p24(73_3_) gained weight, were well-groomed, and appeared to be healthy. Pancreatic tissues from mice immunized orally showed no evidence of pancreatitis.

The present study shows that oral immunization with a CVB4/HIV recombinant induced systemic gag p24-specific T cell responses. Ongoing studies are evaluating the mucosal gag p24-specific immune responses that develop after oral immunization with the CVB4 recombinant. As the search continues for new HIV vaccine strategies capable of inducing systemic and mucosal immune responses, the present proof-of-principle study suggests that live CVB4/HIV recombinants are worth exploring as new vaccine candidates.
